# Peptidomic profiling and molecular dynamics study of bioactive peptides from fermented camel milk: considering the fermentation time dependent proteolysis by *Lactobacillus* and *Saccharomyces* with antidiabetic, antioxidative and anti-inflammatory activities

**DOI:** 10.3389/fnut.2026.1709521

**Published:** 2026-03-10

**Authors:** Prashantkumar Natubhai Padhiyar, Pooja M. Mankad, Krupali Ramanuj, Amar A. Sakure, Arka Bhattacharya, Kanthi Kiran Kondepudi, Bipransh Kumar Tiwary, Zhenbin Liu, Ashish Patel, Subrota Hati

**Affiliations:** 1 Department of Dairy Microbiology, SMC College of Dairy Science, Kamdhenu University, Anand, Gujarat, India; 2 Department of Veterinary Biotechnology, College of Veterinary Science and Animal Husbandry, Kamdhenu University, Anand, Gujarat, India; 3 Gujarat Natural Farming Science University, Halol, Gujarat, India; 4 Department of Agriculture Biotechnology, Anand Agricultural University, Anand, Gujarat, India; 5 Healthy Gut Research Group, Food & Nutritional Biotechnology Division, National Agr-Food and Biomanufacturing Institute, Knowledge City, Mohali, Punjab, India; 6 Department of Microbiology, North Bengal St. Xavier's College, North Bengal University, Siliguri, West Bengal, India; 7 School of Food Science and Engineering, Shaanxi University of Science and Technology, Xi An, China; 8 Department of Animal Genetics and Breeding, College of Veterinary Science and Animal Husbandry, Kamdhenu University, Anand, Gujarat, India

**Keywords:** camel milk, lactic acid bacteria, molecular dynamics, peptide, *Saccharomyce cerevisiae*

## Abstract

This study addresses the growing demand for natural functional foods with therapeutic potential, particularly for managing diabetes, oxidative stress, and inflammation. The aim of this research was to investigate the biofunctional attributes of camel milk fermented with *Limosilactobacillus fermentum* (KGL4) and *Saccharomyces cerevisiae* (WBS2A), with a focus on its antidiabetic, antioxidative, proteolytic, anti-inflammatory, and cytoprotective activities. Fermentation was performed for 0, 12, 36 and 48 h at 30 °C (2% inoculum rate). Fermented camel milk significantly enhanced α-amylase (81.33%) and α-glucosidase (68.37%) inhibition, demonstrating strong antidiabetic potential. Antioxidant activity, as assessed through the ABTS assay, progressively increased with incubation time, reaching a peak of 65.87% at 48 h. Proteolytic activity also rose significantly, attaining a maximum of 6.41 mg/ml (2.5% inoculum concentration at 30 °C/48 h). Chromatographic profiling via RP-HPLC revealed increased bioactivity in 3 kDa permeate (antidiabetic) and 10 kDa retentate (antioxidant) samples, suggesting the role of low-molecular-weight peptides. 2D gel electrophoresis and SDS–PAGE confirmed proteolytic cleavage, revealing the presence of smaller peptide fragments in the fermented samples than in the unfermented controls. Further structural analysis via FTIR and confocal laser scanning microscopy (CLSM) demonstrated secondary structure modifications, including increased β-sheet formation and reduced aggregate size. Molecular docking studies revealed that the identified peptide sequence CCFSSCAMR effectively bound to the human digestive enzymes hBAL, hPAM, and hMGA via hydrogen bonding and hydrophobic interactions, supporting its potential inhibitory function. Additionally, fermented CM displayed strong anti-inflammatory and cytoprotective effects on LPS-stimulated RAW 264.7 macrophages. This study highlights the potential of fermented camel milk as a value-added functional food with significant antidiabetic, antioxidant, anti-inflammatory, and cytoprotective properties. The generation of low-molecular-weight bioactive peptides through targeted microbial fermentation provides a scientific foundation for developing natural, nonpharmacological interventions for metabolic and inflammation-related disorders. These findings support the application of fermented camel milk in functional food and nutraceutical development.

## 1 Introduction

There is growing global interest in functional foods and nutraceuticals derived from natural sources to manage chronic metabolic disorders such as diabetes, oxidative stress, and inflammation. Fermented dairy products, particularly those containing probiotics, have gained attention because of their ability to deliver bioactive compounds with health-promoting effects. Camel milk has emerged as a promising functional food because of its unique nutritional composition and inherent antidiabetic and immunomodulatory properties. However, despite its recognized therapeutic potential, the commercial development and scientific utilization of fermented camel milk remain limited.

Camels (*Camelus dromedarius*), native to desert ecosystems, were domesticated approximately 4,500 years ago in southern Arabia to overcome transportation challenges and have since provided diverse resources, including meat, milk, fur, agricultural labor, tourism, racing, and cultural practices (1, 2). Among these, camel milk (CM) has gained prominence due to its unique nutritional and therapeutic properties, serving as a staple food for nomadic tribes and significantly improving the livelihoods of camel owners. Compared with bovine milk, CM is characterized by lower fat, cholesterol, and lactose contents but is enriched with essential vitamins and minerals, thereby offering improved digestibility and reduced allergenicity through the absence of β-lactoglobulin and reduced β-casein levels. Furthermore, CM contains a wide array of bioactive proteins, such as lactoferrin, lactoperoxidase, lysozyme, immunoglobulin, and peptidoglycan recognition proteins, which collectively confer antimicrobial, anti-inflammatory, and immunomodulatory effects. The presence of nanobodies—small, single-domain antibodies—further enhances immune function, mitigates food allergies, and reduces systemic inflammation. Notably, lactoperoxidase exhibits potent antimicrobial activity against both gram-positive and gram-negative bacteria, reinforcing the therapeutic potential of CM. Empirical evidence highlights its promise in the prevention and management of cancer, anemia, jaundice, and arthritis (3), underscoring its role as a functional food with significant nutritional and medicinal relevance in desert communities. The high zinc content in CM is vital for immune system development and optimal function (4, 5). Camel milk has a total protein content of 31 g/L, with 21.95 g/L total casein. The casein is primarily composed of β-casein (12.09 g/L) and αs1-casein (11.78 g/L), along with smaller amounts of αs2-casein (3.1 g/L) and κ-casein (4.03 g/L), and contains 7.15 g/L whey protein, including >5 g/L α-lactalbumin, and trace amounts of β-lactoglobulin. It is rich in immunoglobulins (0.718 g/L) and serum albumin (3.4 g/L), which are beneficial for immune support and overall health (6). Camel milk is enriched with insulin and insulin-mimetic proteins that regulate B-cell-mediated immune functions and contribute to the amelioration of hyperglycemia (7). Fermentation of CM with LAB enhances its antidiabetic and antioxidative activities. The fermentation process increases the bioavailability of bioactive compounds, which may contribute to the modulation of blood glucose levels and increase insulin sensitivity, making fermented milk a potential therapeutic option for managing diabetes (8, 9). The growing recognition of the health benefits associated with fermented milk products may lead to increased demand within the supply chain. During fermentation, LAB play a vital role in synthesizing various bioactive compounds, including peptides; enzymes such as lipases, amylases, and proteases; and exopolysaccharides (10). LAB strains are known to support human health by modulating the gut microbiota, increasing short-chain fatty acid levels, increasing antioxidant activity, and preserving the integrity of the intestinal lining (11). Furthermore, the bioactive peptides produced by these microbes may help lower diabetes risk (12). Diabetes mellitus is quickly spreading throughout the world. Approximately 9.3% of adults worldwide were estimated to have diabetes in 2019. Approximately 352 million individuals are at risk of T2D according to reports given by the International Diabetes Federation (13). According to health estimates, up to 439 million people globally may have diabetes by 2030. Diabetes was responsible for 15.7% of fatalities in North America and 6% of all deaths in Africa in 2010. Diabetes increases the risk of death in COVID-19 patients, according to studies conducted during the pandemic. To preserve global societal stability, particularly in the face of new infections and illnesses, it is imperative to stop the growth of diabetes (14).

Currently, the development of fermented camel milk faces several challenges. Most studies focus on single-strain LAB fermentation and report only general bioactivity improvements without detailed peptide characterization or mechanistic understanding. The synergistic use of LAB and yeast to increase proteolysis, generate diverse bioactive peptides, and produce functional metabolites remains underexplored. Furthermore, the molecular mechanisms by which fermentation-derived peptides inhibit key metabolic enzymes involved in diabetes and oxidative stress are poorly understood, and few studies integrate bioactivity assays with structural and molecular analyses to validate these effects.

Free radicals, reactive oxygen species (ROS), and reactive nitrogen species (RNS) are produced during normal cellular metabolism. These substances can harm proteins, DNA, and other macromolecules, resulting in oxidative stress and an array of illnesses. The body's natural antioxidants are insufficient to counter this damage, making it essential to include antioxidant-rich foods in the diet. These foods help reduce free radicals and ROS, preventing or minimizing oxidative damage (15, 16). Free radicals pose a significant threat to cellular integrity if they are not efficiently neutralized by endogenous antioxidants. Superoxide dismutase (SOD), a crucial metalloenzyme composed of multiple subunits, is crucial for reducing oxidative stress by promoting the transformation of superoxide radicals into molecular oxygen and hydrogen peroxide via the catalytic mechanism of dismutation. As a primary modulator of the intracellular redox balance, SOD inhibits the excessive presence of detrimental ROS, thereby contributing to the mitigation and control of various disorders associated with oxidative stress in humans (17). The rising demand for natural, health-enhancing products has drawn attention to those rich in biologically active compounds. One innovative application involves the use of such milk-based products to develop specialized wound dressings for chronic and hard-to-heal wounds. These dressings, enriched with bioactive molecules, can enable localized insulin delivery directly at the wound site, potentially accelerating tissue repair. Designed to suit different wound types and individual patient needs, they offer improved therapeutic outcomes. Economically, these advanced dressings may help lower healthcare expenses by accelerating recovery, reducing complications, and increasing patient comfort (18).

Protein hydrolysates and peptides derived from diverse sources, including fish, insects, and plant-based proteins, have demonstrated multifunctional health benefits, such as enzyme inhibition, free radical scavenging, and immune modulation (19–21). These findings underscore the importance of exploring controlled fermentation and enzymatic hydrolysis techniques to enhance the biofunctional properties of food matrices, such as CM, and support the development of scientifically validated functional foods and nutraceuticals.

The purpose of this study was to evaluate the biofunctional enhancement of camel milk through fermentation with lactic acid bacteria (*Limosilactobacillus fermentum* KGL4) and yeast (*Saccharomyces cerevisiae* WBS2A). This study aimed to investigate fermentation time-dependent proteolysis and the generation of low-molecular-weight bioactive peptides resulting from the synergistic action of LAB and yeast. Furthermore, the antidiabetic, antioxidant, anti-inflammatory, and cytoprotective properties of fermented CM were assessed via *in vitro* assays. Structural characterization and molecular docking analyses were performed to elucidate the interactions between the identified peptides and key metabolic enzymes. This study provides a scientific basis for utilizing LAB+yeast cofermentation as an effective strategy to develop camel milk-based functional foods for managing metabolic and oxidative stress-related disorders.

## 2 Materials and methods

### 2.1 Strain

The LAB strain *Limosilactobacillus fermentum* (KGL4, MTCC 25515) and the yeast strain *Saccharomyces cerevisiae* (WBS2A, MG101828) were procured from the Department of Dairy Microbiology, SMC College of Dairy Science, Anand, India.

### 2.2 Sample preparation

Camel milk was hygienically sourced from a small-scale, noncommercial facility situated in Anand, Gujarat, India. The freshly acquired milk was initially filtered through sterile muslin cloth to eliminate any physical contaminants. The mixture was then subjected to autoclave sterilization at 121 °C for 15 min under a pressure of 15 psi to ensure microbial safety. Following thermal treatment, the milk was transferred into presterilized glass containers and stored at a controlled refrigeration temperature of 5 ± 1 °C until further utilization in experimental analyses.

To activate the pure strains, a 2% inoculum was introduced into sterilized camel milk and incubated under specific conditions. Lactic acid bacteria (LAB) were cultured at 30 °C for 24 h, while the yeast was incubated at 25 °C for 2–4 days. For combined fermentation, sterilized CM was inoculated with both *Lactobacillus* and yeast strains at a 2% level. The milk was incubated at 30 °C for different time intervals (12, 24, 36, and 48 h). After each incubation period, the fermented camel milk (FCM) samples were centrifuged at 4,193 × g for 20 min at 4 °C. The supernatants obtained postcentrifugation were then passed through 0.22 μm syringe filters to ensure clarity and sterility and were subsequently subjected to analyses to evaluate their antidiabetic and antioxidant properties.

### 2.3 Evaluation of antidiabetic properties

#### 2.3.1 α-glucosidase inhibition

α-Glucosidase inhibition was measured by reacting the milk supernatant with the enzyme and P-NPG substrate, followed by Na_2_CO_3_ addition and absorbance reading at 405 nm, as described by Shai et al. (22) and Yamaki et al. (23).

#### 2.3.2 α-amylase inhibition

α-Amylase inhibition was assessed by incubating the culture supernatant with α-amylase and starch, followed by the addition of DNSA (3,5-Dinitrosalicylic Acid) reagent, heating, and measuring the absorbance at 540 nm, as described by Telagari et al. (24) and Ademiluyi et al. (25).

### 2.4 Evaluation of antioxidative activity

2,2′-Azino-bis (3-ethylbenzothiazoline-6-sulfonic acid; ABTS) radical scavenging activity was determined by measuring the absorbance at 734 nm via a UV–Vis spectrophotometer, which was based on the protocol described by Sah et al. (26).

### 2.5 Evaluation of proteolysis

Proteolysis was quantified via the OPA (o-Phthaldialdehyde) method as described by Hati et al. (27).

#### 2.5.1 Isolation, purification, and characterization of peptides with antioxidative and antidiabetic effects

The growth conditions were optimized by inoculating cultures at 2.5% inoculation and incubating them at 30 °C for 48 h. The peptide concentration was determined via the OPA assay according to the procedure described by Solanki et al. (28).

#### 2.5.2 Evaluation of SDS–PAGE

Sodium Dodecyl Sulfate–Polyacrylamide Gel Electrophoresis (SDS–PAGE) was conducted in accordance with the methodologies established by Carrasco-Castilla et al. (29) and Laemmli (30) to determine the molecular weights of protein fragments. Electrophoretic separation was carried out via a 12% resolving gel incorporating water-soluble extracts (WSEs) derived from FCM for analysis.

#### 2.5.3 2D gel electrophoresis

Two-dimensional gel electrophoresis was employed to resolve peptide spots from the water-soluble extracts (WSEs) of FCM, adhering to the protocol described by Yang et al. (31), with electrophoretic conditions refined in accordance with the modifications proposed by Panchal et al. (32).

#### 2.5.4 Isoelectric focusing

A total of 125 μg of protein extracted via FCM was applied to a 7 cm IPG strip (pH 3–10, Bio-Rad) for isoelectric focusing. After equilibration in two buffers (10 min each), the strip was rinsed with 1X TGS buffer (30 s) and subjected to SDS–PAGE for 2D analysis via gel preparation methods based on Carrasco–Castilla et al. (29) and Laemmli (30).

#### 2.5.5 Identification and structural characterization of peptides via RPLC/MS

##### 2.5.5.1 Liquid chromatography

Liquid chromatography was performed with an Adhoc column (2.1 × 100 mm, 1.5 μm, India), with the sample compartment maintained at 20 °C and the column temperature set at 40 °C. Chromatographic separation was performed with a binary mobile phase system comprising solvent A (acetonitrile with 0.1% formic acid) and solvent B (ultrapure water). Protein spots isolated via two-dimensional (2D) gel electrophoresis were subjected to in-gel enzymatic digestion via trypsin. The resulting peptide digest was subsequently filtered through a 0.22 μm nylon membrane and introduced into the system in a 20 μl injection volume. Separation was conducted at a constant flow rate of 0.3 ml/min. The gradient elution conditions were meticulously optimized on the basis of the protocol delineated by Khakhariya et al. (33).

Mass spectrometric analysis was carried out via an AB SCIEX QTRAP 4,500 mass spectrometer equipped with an electrospray ionization (ESI) source in conjunction with an ABSCIEX QTRAP 4500 and Ekspert ultra-LC 100 from Eksigent, USA. Peptide profiling of fermented camel milk samples was conducted through reverse-phase liquid chromatography coupled with mass spectrometry (RPLC-MS), employing a scan strategy that incorporated enhanced mass spectra (EMS) followed by enhanced product ion (EPI) scans. The EMS mode was configured to detect ions within a mass–charge (*m*/*z*) range of 350–2,000 Da. The instrument parameters were set as follows: declustering potential (DP) of 80 V, entrance potential (EP) of 10 V, and ion spray voltage of 5,500 V, with a rolling collision energy ramp applied for fragmentation. EPI scans were optimized to detect fragment ions generated under high-energy collision-activated dissociation (CAD), maintaining consistent DP and EP values and covering a m/z range of 100–2,000 Da. To improve peptide detection and identification, an information-dependent acquisition (IDA) approach was utilized, in which the top one to three most intense precursor ions within a 250 mDa mass window were targeted. Additionally, enhanced resolution (ER) scans were applied to assess the isotopic profiles of the observed ions.

##### 2.5.5.2 Analysis of data and peptide identification

The raw mass spectral data of the peptides were analyzed via MASCOT software for peptide identification. The resulting peptide sequences were subsequently cross-referenced with the BIOPEP database to validate their potential antioxidative and antidiabetic bioactivities.

#### 2.5.6 Peptide fractionation via RP-HPLC

Peptides isolated from fermented camel milk (FCM) were characterized via reverse-phase high-performance liquid chromatography (RP-HPLC) on a ShimadzuLC system, Japan. Separation was achieved with a Thermo Fisher Scientific (USA) UMSil C18 (3) analytical column (5 μm particle size, 250 × 4.6 mm dimensions). A 20 μl sample volume was introduced into the system via a precision loop microinjector (HAMILTON Bonaduz AG, Switzerland). The mobile phase consisted of two solvents: Eluent A−0.01% (*v*/*v*) trifluoroacetic acid (TFA) in deionized water—and Eluent B−0.01% (*v*/*v*) TFA in a solvent mixture of 80% acetonitrile and 20% deionized water. The chromatographic run was conducted at room temperature, maintaining a flow rate of 0.25 ml/min.

The gradient elution protocol was programmed as follows: 10% B (0–1 min), 20% B (1–10 min), 25% B (10–15 min), 35% B (15–20 min), 50% B (20–30 min), 60% B (30–33 min), 70% B (33–36 min), 80% B (36–39 min), and a final ramp from 90 to 100% B (39–50 min). Peptide elution was detected at 214 nm via a Shimadzu SPD-20A UV–Visible detector. The number of peptide peaks was quantified in accordance with the procedure described by Khakhariya et al. (34).

#### 2.5.7 Ultrafiltered fractions of FCM through RP-HPLC

Peptides were purified from ultrafiltered fractions via FCM (Fermented camel milk) via RP-HPLC. Fermentation was initiated with a 2.5% inoculum and incubated at 30 °C for 48 h. The samples were subsequently centrifuged at 4,193 × g for 30 min to obtain water-soluble extracts (WSEs). This extract was then processed through ultrafiltration membranes with molecular weight cutoffs of 3 and 10 kDa to fractionate peptides on the basis of size. The antidiabetic efficacy of the resulting fractions was evaluated via α-amylase and α-glucosidase inhibition assays, as detailed in Sections 2.3.1 and 2.3.2, respectively. Furthermore, both the permeate and the retentate were assessed for their antioxidant potential via the ABTS radical scavenging method, following the procedure described in Section 2.4.

#### 2.5.8 Visualization of protein biomolecules via FCM via confocal laser scanning microscopy (CLSM) and Fourier transform infrared (FTIR) spectroscopy

Proteins were conjugated with fluorescein isothiocyanate (FITC) for fluorescent labeling, which was prepared at a 1% concentration using Milli-Q water. Fluorescence was activated by laser excitation at a wavelength of 540 nm. Visualization of the protein structures was performed via a × 63 objective lens.

Functional group characterization of the samples was performed via attenuated total reflectance Fourier transform infrared (ATR-FTIR) spectroscopy, which employs an Alpha spectrometer (Bruker, Germany). Infrared spectra were acquired across a wavenumber range of 4,000–500 cm^−1^, with a spectral resolution of 4 cm^−1^, averaging 24 consecutive scans per sample. The analytical protocol adhered to the methodology outlined by Pipaliya et al. (35).

### 2.6 Anti-inflammatory activity

#### 2.6.1 Cytotoxicity assay

The cells cultured in T25 flasks at 70%−80% confluency were harvested and subsequently seeded into 96-well plates at a density of approximately 1 × 10^5^ cells per well. The plates were incubated for 16 h at 37 °C in a humidified atmosphere containing 5% CO_2_ to facilitate cell adherence. Following incubation, the cells were exposed to varying dilutions of the milk sample and incubated for an additional 24 h under the same conditions.

After treatment, the supernatant was carefully aspirated, and 100 μl of MTT solution (0.5 mg/ml) was added to each well. The plates were then incubated for 4 h at 37 °C in a 5% CO_2_ environment to allow the formation of formazan crystals. The medium was subsequently removed, and 100 μl of dimethyl sulfoxide (DMSO) was added to solubilize the formazan. The absorbance was measured at 570 nm via a Tecan Life Sciences M200 PRO microplate reader to assess cell viability.

#### 2.6.2 Cell viability

RAW264.7 cells were obtained from the National Center for Cell Science (NCCS), Pune, Maharashtra, India. The cells were maintained in high-glucose Dulbecco's modified Eagle's medium (DMEM; Gibco #11965118) supplemented with 10% fetal bovine serum (FBS; Gibco #A5256701) and 1% penicillin–streptomycin (Gibco #15140122). Cultures were incubated at 37 °C in a humidified atmosphere containing 5% CO_2_. Routine subculturing was carried out every 1–2 days in T25 flasks to ensure optimal cell growth and viability.

#### 2.6.3 Reactive oxygen species production

To evaluate total ROS production in RAW264.7 cells after coexposure to cotreatment with LPS (1 μg/ml) and fermented milk, approximately 1 × 10^5^ cells were seeded per well of a 12-well plate and incubated for 24 h at 37 °C with 5% CO_2_. Next, the cells were exposed to LPS (1 μg/ml), fermented milk, or a combination of LPS (1 μg/ml) and fermented milk for the next 24 h. The cells were scraped after incubation and resuspended in 20 μM DCFDA in plain media, followed by a 30 min dark incubation period at 37 °C. A BD FACSAria III flow cytometer in the FITC-A channel was then used to examine the cells.

#### 2.6.4 Estimation of TNF-α, IL-6, and IL-1β cytokine levels

The concentrations of TNF-α, IL-6, and IL-1β in the collected culture supernatants were quantitatively assessed via commercially available ELISA kits (Elabscience, USA), adhering strictly to the manufacturer's recommended protocol.

### 2.7 Molecular docking

The two-dimensional and three-dimensional structures of human bile salt-activated lipase (hBAL), maltase-glucoamylase (hMGA), and pancreatic alpha-amylase (hPAM) were obtained from the Protein Data Bank (PDB) via accession codes 1F6W, 3CTT, and 3BAI, respectively. Prior to molecular docking, structural refinement was performed by removing all water molecules and cocrystallized ligands, followed by the addition of polar hydrogen atoms via AutoDock version 4.2.6 (36). The tertiary structures of the peptides were computationally modeled via the PEPstrMOD server (37).

Active site prediction of the target enzymes was carried out through the PrankWeb server (38), and binding pockets with the highest confidence scores were selected for subsequent docking studies. Protein–peptide interactions were simulated via GalaxyPepDock (39), with the default parameter settings applied. The most energetically favorable docking poses were analyzed to explore key noncovalent interactions, including hydrogen bonds and hydrophobic contacts, between the peptides and the active site residues of the enzymes. Interaction maps were generated and interpreted via LigPlot+, a tool provided by the European Bioinformatics Institute, while the final docked complexes were rendered and visualized via the BIOVIA Discovery Studio Visualizer (Dassault Systèmes).

### 2.8 Statistical analysis

Data analysis was conducted via a two-factor completely randomized design (CRD) to evaluate the effects of individual factors on selected parameters. Statistical significance was determined at the 5% level, in accordance with the methodology outlined by Steel et al. (40). GraphPad Prism version 8.0 (GraphPad Software, Inc. La JOLLA, CA, USA) was used for all the statistical computations. Group comparisons were carried out via one-way analysis of variance (ANOVA), followed by Tukey's *post hoc* test for multiple comparisons. All experiments were performed in triplicate, and differences were deemed statistically significant at *P* < 0.05.

## 3 Results

### 3.1 Antidiabetic antioxidative and proteolytic activities of fermented camel milk

**Figure 1:**
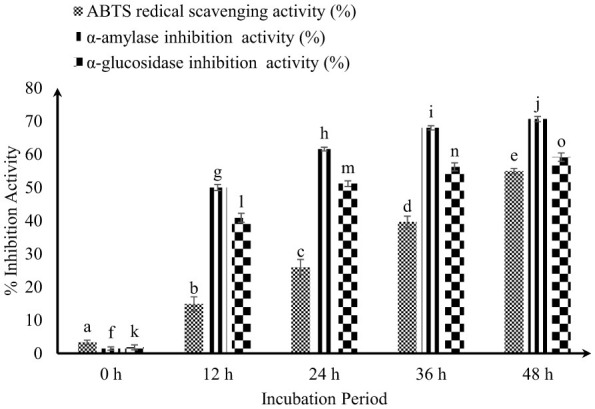
ABTS radical scavenging and antidiabetic activities (%) of fermented camel milk. Values with different superscripts differ significantly (*P* < 0.05), ABTS radical scavenging and antidiabetic activities (%) Mean ± SD of three replicate experiments (*n* = 3).

Figure 1 illustrates the antidiabetic efficacy of FCM inoculated with KGL4 + WBS2A. A marked and statistically significant increase (*P* < 0.05) in all evaluated antidiabetic parameters was observed with prolonged fermentation duration. Notably, the highest inhibitory activities against α-glucosidase and α-amylase were observed after 48 h of incubation, with inhibition rates reaching 65.77 and 80.64%, respectively. This enhanced enzyme inhibition at 48 h can be attributed to the optimal progression of fermentation, during which microbial proteolytic systems actively hydrolyze milk proteins into low-molecular-weight bioactive peptides. These peptides are known to possess strong affinity toward carbohydrate-hydrolyzing enzymes because of the presence of specific amino acid sequences capable of interacting with the catalytic and peripheral binding sites of α-glucosidase and α-amylase. The observed inverse relationship between incubation duration and enzyme activity indicates that increased fermentation time leads to the progressive accumulation of inhibitory peptides and metabolites, resulting in reduced enzymatic hydrolysis of carbohydrates. However, beyond the optimal incubation period, excessive proteolysis may further degrade these bioactive peptides into smaller, less active fragments, potentially reducing their inhibitory efficacy.

Figure 1 presents the antioxidant activity of FCM over different incubation periods at 30 °C. The antioxidative activity of FCM progressively increased with increasing incubation time. At 0 h, the inhibition was 4.62 ± 0.13%, which significantly increased to 22.45 ± 1.33% at 12 h, 38.34 ± 1.66% at 24 h, 48.95 ± 1.88% at 36 h, and reached a maximum of 65.87 ± 1.50% after 48 h of fermentation. This time-dependent increase suggests that prolonged fermentation promotes the release of bioactive peptides with antioxidative potential. As fermentation progresses, protein unfolding and peptide bond cleavage expose amino acid residues such as histidine, tyrosine, tryptophan, cysteine, and proline, which are known to contribute to antioxidant activity through hydrogen atom donation, electron transfer, and metal ion chelation. These peptides can effectively neutralize reactive oxygen species (ROS), thereby reducing oxidative stress.

**Figure 2:**
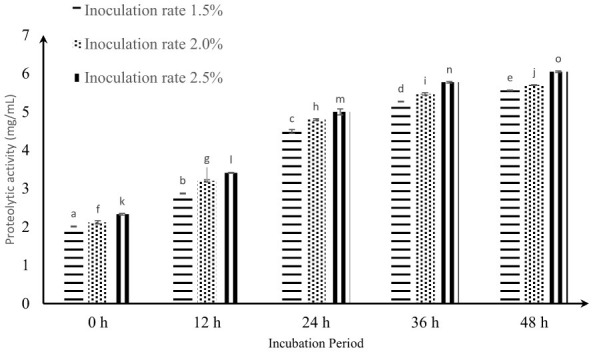
Effect of inoculation rates and incubation period on proteolytic activity (mg/ml) of fermented camel milk. Values with different superscripts differ significantly (*P* < 0.05), Proteolytic activity (mg/ml) Mean ± SD of three replicate experiments (*n* = 3).

Figure 2 shows the proteolysis in CM fermented with the *KGL4* + *WBS2A* strains under various inoculum concentrations and incubation periods. At an initial inoculation level of 1.5%, proteolytic activity was recorded at 2.32 ± 0.03 mg/ml (0 h), which progressively increased, reaching a peak value of 6.41 ± 0.01 mg/ml (48 h at a 2.5% rate). The results indicate that both increased inoculum levels (1.5%, 2.0%, and 2.5%) and prolonged incubation time significantly (*P* < 0.05) increased proteolytic activity. A significant interaction effect was also observed between the inoculation concentration and fermentation duration. The highest proteolytic activity (6.41 ± 0.01 mg/ml) was achieved (48 h at 2.5%), notably surpassing the activity levels observed at lower inoculation rates. However, proteolytic activity increased steadily with fermentation time and inoculum concentration, indicating increased microbial enzymatic activity. The gradual increase in proteolysis can be attributed to the activation of extracellular and intracellular proteases produced by lactic acid bacteria and yeast during fermentation, which hydrolyze caseins and whey proteins into smaller peptides and free amino acids.

### 3.2 Ultrafiltered fractions of FCM through RP-HPLC

**Figure 3:**
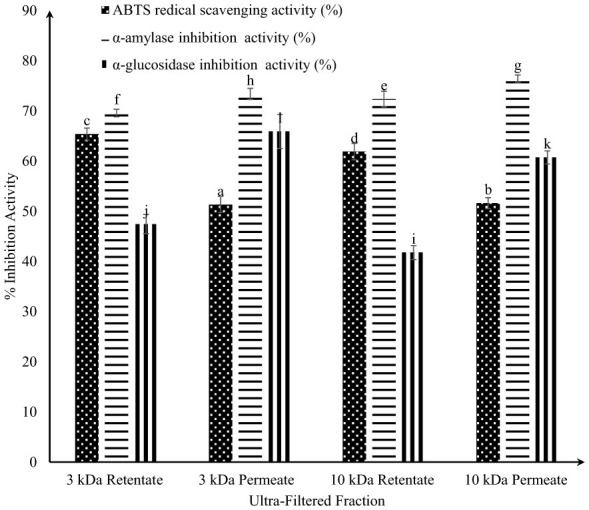
ABTS radical scavenging and antidiabetic activities of ultra-filtered fractions (3 kDa and 10 kDa permeate and retentate) from fermented camel milk. Values with different superscripts differ significantly (*P* < 0.05), ABTS radical scavenging and antidiabetic activities (%) Mean ± SD of three replicate experiments (*n* = 3).

Peptides from the 3 kDa and 10 kDa permeate and retentate fractions were profiled via RP-HPLC under optimized camel milk fermentation conditions (2.5% inoculum, 48 h at 30 °C). As shown in Table 1, peptide fractions were categorized as < 3 kDa, >3 kDa, < 10 kDa, and >10 kDa. Comparative chromatographic profiles (Figures S1–S6) clearly demonstrated an increase in peptide diversity and concentration postfermentation, with fermented samples displaying distinct peaks between 5 and 47 min, unlike the unfermented samples, which presented primarily intact protein signals. The ultrafiltered fractions of fermented camel milk exhibited substantial antioxidant and antidiabetic activities, as presented in Figure 3. Notably, the 3 kDa permeate showed maximum α-amylase inhibition (84.20%), the 10 kDa permeate exhibited strong α-glucosidase inhibition (67.04%), and the 10 kDa retentate displayed the greatest antioxidant activity (66.31%; as shown in Figure 3), indicating robust biofunctional potential. The antidiabetic and antioxidative activities of the ultrafiltered fractions are shown in Figure 3.

**Table 1:** Amino acid sequences obtained from camel milk fermented with M9+WBS2A with peptide ranking score.

**Sequences**	**Peptide ranking score**	**Mol. wt**.	**Prediction**	**Retention time**	**Net charge at pH 7**	**Hydro-phobicity**	**Hydro-pathicity**	**Hydro-philicity**
EYGLFQINNK	0.214334	1,225.52	Non-toxin	13.265	0	−0.16	−0.85	−0.18
KILDKEGIDYWLAHK	0.428301	1,829.36	Non-toxin	30.167	0.5	−0.16	−0.64	0.27
MMSLVSLLLVGILFPTIQAKQFTK	0.97371	2,679.75	Non-toxin	1.7172	2	0.12	1.16	−0.73
DILKEDMPSQRYLEELHR	0.125535	2,272.83	Non-toxin	2.3015	−1.5	−0.37	−1.28	0.73
YLEELHR	0.117655	959.17	Non-toxin	8.2718	−0.5	−0.33	−1.2	0.37
DQKLIPR	0.463075	869.13	Non-toxin	5.9379	1	−0.44	−1.24	0.8
VMDVPKTKETIIPK	0.151499	1,599.17	Non-toxin	2.4189	1	−0.17	−0.29	0.45
PSGFQLFGSPAGQKDLLFK	0.797903	2,037.63	Non-toxin	14.353	1	−0.04	−0.14	−0.18
YPSYGINYYQHR	0.432274	1,560.86	Toxin	10.516	1.5	−0.24	−1.52	−0.65

### 3.3 Protein profiling through SDS–PAGE and 2D *gel* electrophoresis

**Figure 4:**
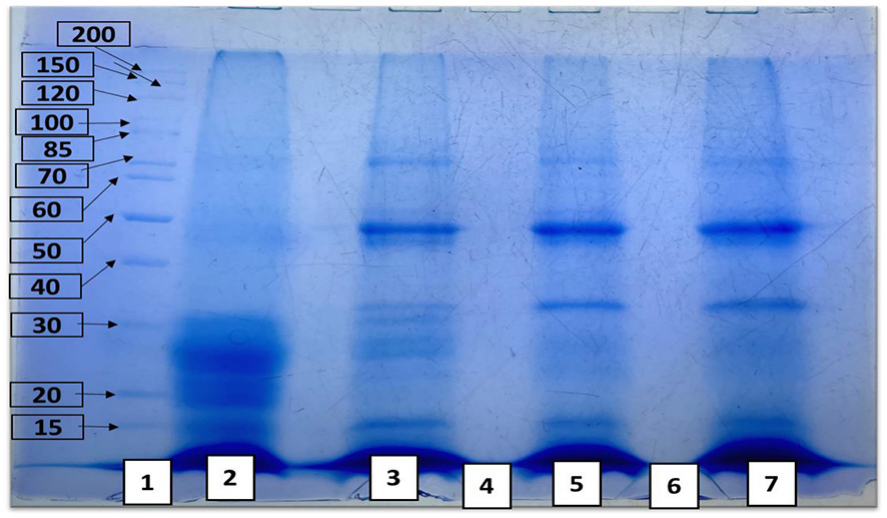
Protein and peptide profile of camel milk fermented with M9+WBS2A revealed by SDS-PAGE (1: Protein ladder, 2: Unfermented camel milk, 3: Fermented camel milk, 4: 3 kDa permeate, 5: 3 kDa retentate, 6: 10 kDa permeate, 7: 10 kDa retentate).

Camel milk fermented with the microbial consortia *KGL4* and *WBS2A* was evaluated via SDS–PAGE, which employs a molecular weight marker (10–200 kDa). In unfermented camel milk, a band was primarily observed between 15 and 30 kDa, indicating the presence of intact, native proteins. Following fermentation, the camel milk sample presented an expanded range of protein bands between 15 and 85 kDa, suggesting that proteolysis had occurred due to the enzymatic activity of the microbial strains. These results are visually represented in Figure 4.

**Figure 5:**
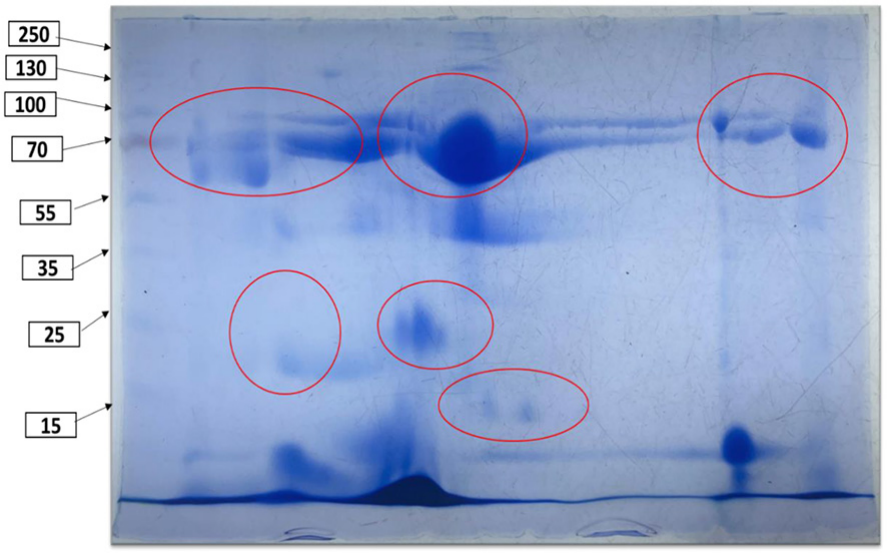
2D gel electrophoresis of camel milk fermented with M9+WBS2A.

2D-PAGE analysis via FCM revealed 33 distinct protein spots, primarily within the 15–70 kDa range, indicating active proteolysis and the generation of short-chain peptides, as illustrated in Figure 5.

### 3.4 Peptide identification and structural analysis via RPLC–MS

Trypsin-digested protein spots from 2D-PAGE of FCM were analyzed via RP–LC–MS. Spectral data (EMS to EP1 scans) were processed via PeakView software and matched against camel milk protein databases. The identified peptide masses were evaluated through Mascot and cross-referenced with the Swiss-Prot (Table 2) and BIOPEP databases to identify potential antidiabetic and antioxidative peptides. Peptides were selected on the basis of toxicity and peptide Ranker scores, as detailed in Tables 3 and 4. Figure S7A, B shows the total ion chromatogram of fermented camel milk acquired through LC–MS analysis via EMS to EPI scanning.

**Table 2:** Amino acid sequences with antioxidant activity obtained from camel milk fermented with M9+WBS2A searched on BIOPEP database.

**Sequences**	**ID**	**Matched sequences**	**Molecular mass**	**Source**	**References**
EYGLFQINNK	10101	YGLF	498.24	Soybean protein	Amigo et al. (55)
KILDKEGIDYWLAHK	7793	AHK	354.20	Egg protein	Mine (56)
	7886	AH	226.10	Soybean protein	Chen et al. (57)
MMSLVSLLLVGILFPTIQAKQFTK	9086	MM	280.09	protein hydrolysate	Anna et al. (58)
DILKEDMPSQRYLEELHR	3305	LH	268.15	Soybean protein	Chen et al. (57)
	7888	EL	260.13	**Casein**	Suetsuna et al. (47)
	7999	LHR	424.25	**Synthetic**	Saito et al. (53)
	8217	LK	259.18	Egg white protein	Huang et al. (59)
	10051	RY	337.17	Soybean protein	Amigo et al. (55)
	10052	RYL	450.25	Soybean protein	Amigo et al. (55)
	10733	YL	294.15	Perilla	Yang et al. (62)
YLEELHR	3305	LH	268.15	Soybean protein	Chen et al. (57)
	7888	EL	260.13	Casein	Suetsuna et al. (47)
	7999	LHR	424.25	**Synthetic**	Saito et al. (53)
	10733	YL	294.15	Perilla	Yang et al. (62)
VMDVPKTKETIIPK	10771	PK	243.15	Soybean protein	Wang et al. (60)

**Table 3:** Amino acid sequences with antidiabetic activity obtained from camel milk fermented with M9+WBS2A searched on BIOPEP database.

**Sequences**	**ID**	**Matched sequences**	**Molecular mass**	**Source**	**References**
EYGLFQINNK	8561	GL	188.22	**Milk protein**	Nongonierma et al. (41)
	8777	EY	310.30	Soy protein hydrolysates	Lan et al. (42)
	8779	FQ	293.31	Soy protein hydrolysates	Lan et al. (42)
	8804	IN	245.13	Soy protein hydrolysates	Lan et al. (42)
	8847	NN	246.09	Soy protein hydrolysates	Lan et al. (42)
	8873	QI	259.30	Soy protein hydrolysates	Lan et al. (42)
	8936	YG	238.09	Soy protein hydrolysates	Lan et al. (42)
KILDKEGIDYWLAHK	3175	LA	202.25	Rat intestinal brush border membrane	Bella et al. (61)
	8677	WL	317.38	**Milk protein**	Nongonierma et al. (41)
	8761	AH	226.23	Soy protein hydrolysates	Lan et al. (42)
	8770	EG	204.18	Soy protein hydrolysates	Lan et al. (42)
	8785	GI	188.22	Soy protein hydrolysates	Lan et al. (42)
	8802	IL	244.32	Soy protein hydrolysates	Lan et al. (42)
	8808	KE	275.1476	Soy protein hydrolysates	Lan et al. (42)
	8812	KI	259.34	Soy protein hydrolysates	Lan et al. (42)
	8947	YW	367.39	Soy protein hydrolysates	Lan et al. (42)
MMSLVSLLLVGILFPTIQAKQFTK	3182	LL	244.32	Rat intestinal brush border membrane	Bella et al. (61)
	8506	FP	262.30	Rice bran	Hatanaka et al. (43)
	8560	SL	218.24	**Milk protein**	Nongonierma et al. (41)
	8785	GI	188.22	Soy protein hydrolysates	Lan et al. (42)
	8802	IL	244.32	Soy protein hydrolysates	Lan et al. (42)
	8805	IQ	259.30	Soy protein hydrolysates	Lan et al. (42)
	8825	LV	230.30	Soy protein hydrolysates	Lan et al. (42)
	8833	MM	280.40	Soy protein hydrolysates	Lan et al. (42)
	8863	PT	216.23	Soy protein hydrolysates	Lan et al. (42)
	8867	QA	217.22	Soy protein hydrolysates	Lan et al. (42)
	8870	QF	293.13	Soy protein hydrolysates	Lan et al. (42)
	8903	TI	232.27	Soy protein hydrolysates	Lan et al. (42)
	8904	TK	247.15	Soy protein hydrolysates	Lan et al. (42)
	8918	VG	174.19	Soy protein hydrolysates	Lan et al. (42)
	8926	VS	204.22	Soy protein hydrolysates	Lan et al. (42)
DILKEDMPSQRYLEELHR	3171	MP	246.32	Rice bran	Hatanaka et al. (43)
	8794	HR	311.33	Soy protein hydrolysates	Lan et al. (42)
	8802	IL	244.32	Soy protein hydrolysates	Lan et al. (42)
	8808	KE	275.14	Soy protein hydrolysates	Lan et al. (42)
	8820	LH	268.31	Soy protein hydrolysates	Lan et al. (42)
	8862	PS	202.20	Soy protein hydrolysates	Lan et al. (42)
	8940	YL	294.15	Soy protein hydrolysates	Lan et al. (42)
YLEELHR	8794	HR	311.33	Soy protein hydrolysates	Lan et al. (42)
	8820	LH	268.31	Soy protein hydrolysates	Lan et al. (42)
	8940	YL	294.15	Soy protein hydrolysates	Lan et al. (42)
DQKLIPR	8501	IP	228.28	Rice bran	Hatanaka et al. (43)
	8768	DQ	261.09	Soy protein hydrolysates	Lan et al. (42)
	8821	LI	244.32	Soy protein hydrolysates	Lan et al. (42)
VMDVPKTKETIIPK	3181	VP	214.26	Rice bran	Hatanaka et al. (43)
	8501	IP	228.28	Rice bran	Hatanaka et al. (43)
	8774	ET	248.23	Soy protein hydrolysates	Lan et al. (42)
	8801	II	244.17	Soy protein hydrolysates	Lan et al. (42)
	8808	KE	275.14	Soy protein hydrolysates	Lan et al. (42)
	8816	KT	247.15	Soy protein hydrolysates	Lan et al. (42)
	8858	PK	243.30	Soy protein hydrolysates	Lan et al. (42)
	8903	TI	232.27	Soy protein hydrolysates	Lan et al. (42)
	8904	TK	247.15	Soy protein hydrolysates	Lan et al. (42)
	8923	VM	248.11	Soy protein hydrolysates	Lan et al. (42)
PSGFQLFGSPAGQKDLLFK	3179	PA	186.20	Rat intestinal brush border membrane	Bella et al. (61)
	3182	LL	244.32	Rat intestinal brush border membrane	Bella et al. (61)
	8505	SP	202.20	Rice bran	Hatanaka et al. (43)
	8760	AG	146.14	Soy protein hydrolysates	Lan et al. (42)
	8779	FQ	293.31	Soy protein hydrolysates	Lan et al. (42)
	8782	GF	222.23	Soy protein hydrolysates	Lan et al. (42)
	8862	PS	202.20	Soy protein hydrolysates	Lan et al. (42)
	8874	QL	259.30	Soy protein hydrolysates	Lan et al. (42)
	3179	PA	186.20	Rat intestinal brush border membrane	Bella et al. (61)
YPSYGINYYQHR	8521	YP	278.30	Rice bran	Hatanaka et al. (43)
	8785	GI	188.22	Soy protein hydrolysates	Lan et al. (42)
	8794	HR	311.33	Soy protein hydrolysates	Lan et al. (42)
	8804	IN	245.13	Soy protein hydrolysates	Lan et al. (42)
	8853	NY	295.11	Soy protein hydrolysates	Lan et al. (42)
	8862	PS	202.20	Soy protein hydrolysates	Lan et al. (42)
	8872	QH	283.12	Soy protein hydrolysates	Lan et al. (42)
	8897	SY	268.10	Soy protein hydrolysates	Lan et al. (42)
	8936	YG	238.09	Soy protein hydrolysates	Lan et al. (42)
	8943	YQ	309.31	Soy protein hydrolysates	Lan et al. (42)
	8948	YY	344.13	Soy protein hydrolysates	Lan et al. (42)

**Table 4:** Characterization of < 3 kDa, >3 kDa, < 10 kDa, >10 kDa peptides generated from fermented camel milk by RP-HPLC analysis.

**Milk**	**Sample**	**Number of peaks**	**Retention time (min)**
Camel milk (M9+WBS2A)	< 3 kDa	43	6.3–46.67
	>3 kDa	34	6.7–46.68
	< 10 kDa	42	6.4–46.69
	>10 kDa	36	6.7–46.69

In this study, the peptide CCFSSCAMR, obtained from soy protein hydrolysates, was aligned with previously reported antidiabetic peptide fragments MR (39), as listed in the BIOPEP database. These sequence matches highlight its potential antidiabetic bioactivity. In this study, the peptide TDVMPQWW was identified and found to correspond with several peptide fragments previously reported for antidiabetic potential, as listed in the BIOPEP database. These include MP (40) (rice bran), WW (41) (milk protein), PQ (39) (soy protein hydrolysates), QW (39) (soy protein hydrolysates), TD (39) (soy protein hydrolysates), and VM (soy protein hydrolysates). These sequence matches reinforce the potential antidiabetic bioactivity of TDVMPQWW.

The sequence KLLILTCLVAVALAR, identified from camel milk fermented with KGL4+WBS2A, was similar to those of the peptides LTC and CLV (42), as well as LT (43). Similarly, the peptide YLEELHRLNKYK matched previously reported peptides such as LH (41), EL (44), LHR (45), and YL (46), as listed in the BIOPEP database. These peptide alignments support the antioxidant potential of the sequences identified in this study.

### 3.5 Visualization of protein biomolecules via FCM via confocal laser scanning microscopy and Fourier transform infrared spectroscopy

Fermented camel milk displayed more complex protein structures and larger protein aggregates, which was attributed to the action of the *KGL4* and *WBS2A* strains. These microbial cultures promote the conversion of native milk proteins into various polymeric forms during fermentation. As shown in Figure S8, membrane filtration was performed using molecular weight cutoffs (3 and 10 kDa), permeate and retentate. Notably, the unfermented samples presented larger particle sizes than the FCM samples did, indicating that fermentation contributed to the breakdown of higher-molecular-weight proteins. In camel milk fermented with the *KGL4* + *WBS2A* strains, the 3- and 10-kDa permeate fractions contained smaller peptides, whereas the corresponding retentate fractions retained larger protein fragments.

**Figure 6:**
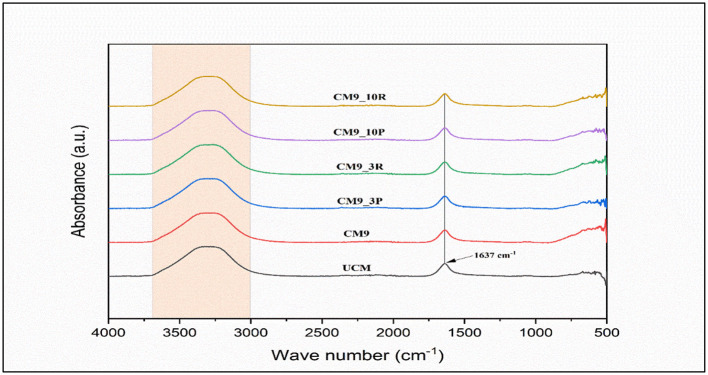
Fourier transform-infrared spectroscopy (FTIR) of camel milk fermented with M9+WBS2A (USM—Unfermented camel milk, CM9—camel milk fermented with M9+WBS2A, CM9-3P−3 kDa permeate, CM9-3R−3 kDa retentate, CM9-10P−10 kDa permeate, CM9-10R−10 kDa retentate).

The FTIR spectrum of CM fermented with the *KGL4*+*WBS2A* strain revealed significant changes in the protein secondary structure, as shown in Figure 6. A prominent increase in absorbance was observed at 1,637 cm^−1^ in the amide I region, which was attributed to the C=O stretching vibrations of peptide bonds. This shift, compared with that of unfermented camel milk, indicates proteolytic modifications to the protein structure during fermentation. Additionally, an increase in the 1,620–1,640 cm^−1^ range was noted, corresponding to β-sheet structures, which were particularly evident in the 3 kDa and 10 kDa permeate and retentate fractions. Deconvolution and curve fitting of the raw FTIR data were performed via Origin software, allowing quantification of secondary structural elements. The analysis revealed that the β-sheet content was significantly greater in the fermented samples than in the unfermented samples, whereas the α-helix structures were reduced, further supporting the hypothesis of extensive proteolysis. These findings are consistent with the interpretations described by Barth (54), who noted that the amide I region of the FTIR spectrum is highly sensitive to backbone conformations and can be used to monitor secondary structural transitions in proteins. These spectral transitions reflect the unfolding and reorganization of protein chains due to microbial activity, leading to the release of lower-molecular-weight bioactive peptides. The 2D secondary structure profile confirmed that fermentation with *KGL4*+*WBS2A* induced substantial structural rearrangements in CM proteins, increasing their functional potential.

### 3.6 Anti-inflammatory activity of FCM in RAW 264.7 cells

**Figure 7:**
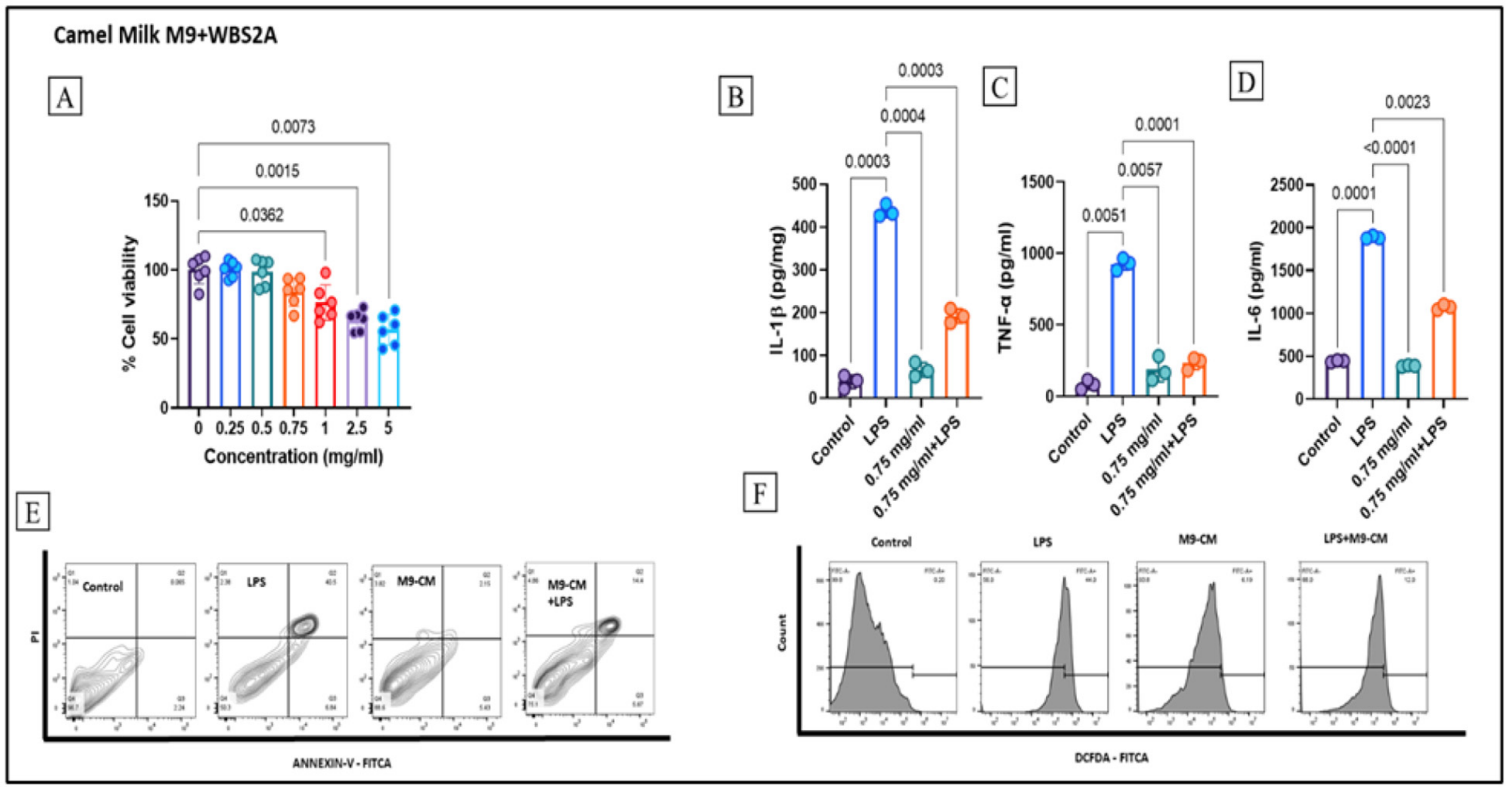
Anti-inflammatory activity and ROS of camel milk fermented with M9+WBS2A, **(A)** cell viability in RAW264.7 cells, **(B)** IL-1β, **(C)** TNF-α, and **(D)** IL-6 production; **(E)** Apoptotic cell population **(F)** ROS production, Data were expressed as mean ± S.E.M and analyzed by one-way ANOVA followed by Tukey's *post hoc* test.

The viability of RAW 264.7 cells exposed to CM fermented with the KGL4+WBS2A strain was evaluated over a range of concentrations, as shown in Figure 7A. At lower doses (0.25 and 0.5 mg/ml), the fermented milk maintained nearly 100% cell viability and showed no cytotoxic effects. In contrast, concentrations between 0.75 and 5 mg/ml produced a progressive decline in viability, reflecting a dose-dependent cytotoxic response. These findings indicate that fermented CM remains biocompatible at relatively low concentrations, whereas relatively high doses may induce mild cytotoxicity in RAW 264.7 cells.

The inflammation-suppressing potential was assessed in RAW 264.7 macrophages stimulated with LPS. The administration of 0.75 mg/ml FCM markedly attenuated the expression of the pivotal proinflammatory cytokines TNF-α, IL-1β, and IL-1β, as illustrated in Figures 7B–D. These findings substantiate the capacity of fermented milk to effectively modulate and suppress inflammatory signaling pathways.

Apoptotic responses were further analyzed via the Annexin V/PI assay. LPS stimulation increased the late apoptotic cell population to 40.5%, indicating inflammation-induced stress. Notably, treatment with fermented CM reduced this percentage to 7.21%, as shown in Figure 7E, indicating strong cytoprotective activity.

To assess the antioxidant effects, the intracellular ROS levels were measured. LPS stimulation alone increased the level of ROS to 44%, whereas treatment with fermented camel milk significantly reduced the level of ROS to 10.8% (Figure 7F), indicating that the high antioxidative activity of the fermented milk was due to the release of bioactive components during fermentation.

Taken together, these findings provide compelling evidence that when fermented with specific strains, camel milk has significant anti-inflammatory, antioxidant, and cytoprotective properties. By downregulating proinflammatory cytokines, reducing oxidative stress, and minimizing apoptosis, fermented camel milk shows promising therapeutic potential for mitigating inflammation-related cellular damage.

### 3.7 Molecular docking of bioactive peptides

This study examined the interactions between the peptide CCFSSCAMR from fermented camel milk and the active sites of hBAL (1F6W), hPAM (3BAI), and hMGA (3CTT). Molecular docking results revealed the peptide's capacity to bind near the catalytic sites of these enzymes, engaging in both hydrogen bonding and nonpolar interactions with essential amino acid residues.

**Figure 8:**
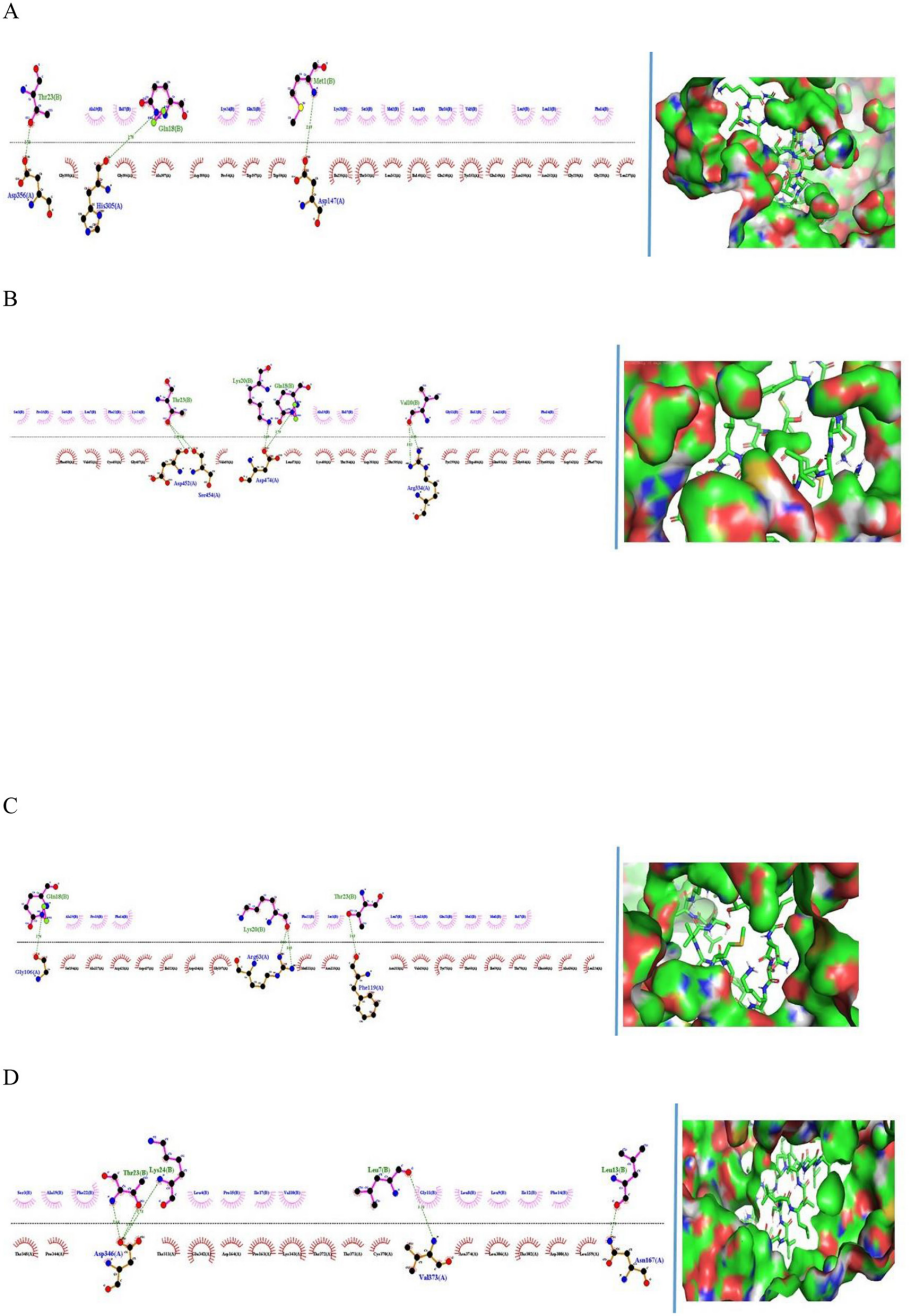
2D and 3D representation of binding interactions between peptide MMSLVSLLLVGILFPTIQAKQFTK and **(A)** human pancreatic alpha-amylase (hPAM) **(B)** human maltase-glucoamylase (hMGA), **(C)** human bile salt activated lipase (hBAL) and **(D)** human angiotensin-converting enzyme (hACE), respectively. Peptidyl residues are denoted as blue color and receptor residues are denoted black color; hydrogen bonds are marked in dotted lines.

The results of the GalaxyPepDock analysis, including protein structure similarity, interaction similarity and estimated accuracy scores for each protein–peptide complex and residues involved in hydrogen bonding, are summarized in Table 5. Among the three enzymes analyzed, hBAL demonstrated the highest affinity for CCFSSCAMR, suggesting a strong and stable interaction. Comparatively weaker binding was observed with hPAM and hMGA. Structural insights obtained through GalaxyPepDock confirmed the quality of the docking, highlighting interaction similarity scores and hydrogen-bonding residues. These findings suggest that CCFSSCAMR has potential as an inhibitory peptide, especially for bile salt-activated lipases. The 2D and 3D structural representations of the peptide CCFSSCAMR are illustrated in Figures 8A–D, respectively.

**Table 5:** HADDOCK score and Hydrogen bonding residues of enzymes against peptide.

**PDB ID**	**HADDOCK scores**	**Residues involved in hydrogen bonds**
3CTT	−107.1 +/−3.7	Asp452; Ser454; Asp474; Arg334
1F6W	−102.6 +/−3.8	Gly106; Arg63; Phe119
3BAI	−113.3 +/−3.3	Asp356; His305;Asp147
1O8A	−3.3 +/−8.5	Asp346; Val373

## 4 Discussion

Recent investigations have emphasized the therapeutic potential of sheep and camel milk, particularly its anti-inflammatory, antidiabetic, and antioxidant activities. These bioactivities are largely attributed to the presence of bioactive peptides, fatty acids, vitamins, and minerals generated or released during fermentation and digestion. These properties not only enhance the nutritional profile of these milks but also suggest their potential role as functional foods in the prevention and management of chronic metabolic disorders, including diabetes and inflammation-associated conditions.

The use of *Lacticaseibacillus paracasei* (M11) with *Saccharomyces cerevisiae* (WBS2A) significantly enhanced the antidiabetic potential of fermented buffalo and CM during incubation. The fermentation period, particularly between 12 and 48 h at 37 °C, had a notable effect on the inhibitory activities related to key digestive enzymes. In buffalo milk, the inhibition of α-glucosidase, α-amylase, and pancreatic lipase increased progressively, reaching 59.95, 79.14, and 63.71%, respectively, after 48 h (33). Similarly, fermented camel milk demonstrated improved inhibitory effects, with α-glucosidase, α-amylase, and lipase inhibition levels reaching 64.55, 81.66, and 69.83%, respectively, by the end of the incubation. This finding suggests that the inhibition increases with incubation time, indicating enhanced antidiabetic potential as fermentation progresses.

Inhibition (α-glucosidase) of FCM using *Lacticaseibacillus paracasei* (M11) was evaluated at various time points during incubation at 37 °C. A progressive increase in inhibition was observed over time, beginning at 47.10 ± 3.80% after 12 h, increasing to 55.62 ± 0.53% at 24 h, 58.14 ± 0.64% at 36 h, and reaching 59.62 ± 0.83% after 48 h. This gradual increase indicates that prolonged fermentation significantly increased the α-glucosidase inhibition effect. These results align with our findings, reinforcing the conclusion that extended fermentation improves the antidiabetic potential of FCM (8).

The antidiabetic efficacy of FCM and Gir cow milk was evaluated following inoculation with selected lactic acid bacteria (LAB) and yeast strains. After 48 h of fermentation at 30 °C, the addition of camel milk had pronounced inhibitory effects on key carbohydrate-hydrolyzing enzymes, resulting in α-amylase and α-glucosidase inhibition levels of 76.54 and 60.06%, respectively. In parallel, fermented Gir cow milk resulted in α-amylase inhibition of 84.41% and α-glucosidase inhibition of 37.10%. These outcomes align with our observations, wherein prolonged incubation markedly enhanced the inhibitory activities against both α-amylase and α-glucosidase (48). These results support the conclusion that prolonged fermentation enhances the antidiabetic potential of milk by promoting the production of bioactive peptides capable of modulating enzymatic activity.

According to Patel et al. (49), camel milk is valued for its nutritional and medicinal benefits, which promote human health. Their study evaluated the antioxidant properties of FCM with *Lactobacillus plantarum* KGL3A via the ABTS assay. The findings revealed a gradual increase in antioxidant potential as fermentation progressed. Initially, at 0 h, the activity was low, but it steadily increased throughout fermentation. The ABTS scavenging activity reached 62.19% (48 h at 37 °C), demonstrating a notable increase in antioxidant capacity over time. These results indicate that prolonged fermentation contributes to the generation of functional compounds that increase the antioxidant potential of camel milk. A similar pattern was evident in our data, as ABTS activity significantly increased with increasing incubation time.

Mehra et al. (50) reported that the maximum ABTS radical scavenging activity after 12 h of fermentation using various bacterial strains of *Lactobacillus delbrueckii* subsp. *bulgaricus* was 61.84 ± 0.34%, whereas *Streptococcus thermophilus* presented the minimum value of 43.60 ± 0.40%. In contrast, our study demonstrated a continuous and significant increase in ABTS activity up to 48 h at 30 °C. This extended incubation with the *KGL4* + *WBS2A* consortium resulted in a markedly greater antioxidant capacity, suggesting that prolonged fermentation enhances the release of bioactive peptides, thereby improving the overall antioxidative potential of fermented camel milk.

The progression of proteolysis of camel milk fermented with *Lacticaseibacillus rhamnosus* with *Saccharomyces cerevisiae* increased from 2.43 ± 0.07 mg/ml (0 h) with a 1.5% inoculation level to a peak of 6.07 ± 0.04 mg/ml (48 h at a 2.5% inoculum). The results revealed a statistically significant increase in proteolysis as both fermentation time and inoculum concentration increased. Moreover, a notable interaction between fermentation duration and inoculation level further amplified the proteolytic response. The maximum enzymatic activity, observed at 6.07 mg/ml (48 h at 2.5%), was substantially greater than that recorded at lower inoculation levels of 1.5% and 2.0% (48). A comparable pattern emerged in our investigation, wherein camel milk fermented with a Lactobacillus–yeast consortium exhibited markedly elevated proteolytic activity.

RP-HPLC chromatographic analysis of ultrafiltered fractions from both fermented and unfermented samples of camel milk and Gir cow milk fermented with the KGL4 and WBS2A strains revealed a noticeable increase in peptide content following fermentation. Unlike unfermented milk, which primarily consists of intact proteins, fermented milk samples displayed distinct protein fragments, with notable peaks detected within retention times of 10–20 and 25–45 min, indicating proteolysis. Among the different molecular weight fractions, the 10 kDa retentate demonstrated the maximum ABTS radical scavenging capacity. However, these antioxidative effects were more pronounced in FCM (69.31%) than in its Gir cow counterpart (51.85%). Similarly, the 3 kDa permeate fractions from both milk types presented the strongest antidiabetic potential. Fermented camel milk showed superior enzyme inhibition, exhibiting α-amylase (81.33%) and α-glucosidase (68.37%) inhibition, whereas fermented Gir cow milk exhibited corresponding inhibition rates of 78.25 and 45.03%, respectively (48). A similar trend was observed in our data, where the 10 kDa retentate showed the maximum antioxidative properties (ABTS assay), and the 3 kDa permeate exhibited the strongest antidiabetic potential. These findings suggest that peptides of lower molecular weight are more effective at exhibiting antidiabetic activity.

The antioxidative properties of whey fractions from FCM and bovine milk were determined via the ABTS assay. Both milk types were fermented with *Lactobacillus rhamnosus* PTCC 1,637, and the water-soluble extracts (WSEs) were fractionated via ultrafiltration (UF) membranes with molecular weight cutoffs of 10, 5, and 3 kDa. Compared with the lower-molecular-mass peptides, the 5–10 kDa peptide fractions presented greater antioxidative potential in both milk types. The Trolox equivalent antioxidant capacity (TEAC) values for the 5–10 kDa fractions ranged from 0.11041 to 0.74535 mM in fermented bovine milk and from 0.84408 to 1.73788 mM in fermented camel milk. Notably, peptide fractions derived from camel milk fermented with *L. rhamnosus* displayed greater antioxidative potential than those from bovine milk (51). This finding aligns with our observations, in which camel milk fermented with a lactic acid bacteria–yeast consortium demonstrated markedly elevated antioxidant capacity, with the 10 kDa retentate showing the most pronounced ABTS radical scavenging activity.

WSEs from both fermented and unfermented buffalo and camel milk fermented with the M11+WBS2A and KGL4+WBS2A strains. The unfermented samples presented a broader range and greater number of protein bands, reflecting the presence of intact proteins. In contrast, the fermented milk samples presented a narrower distribution of bands, suggesting substantial proteolysis induced by the microbial cultures. In fermented samples, protein bands appeared within the 10–75 kDa range, whereas unfermented samples presented bands from 10–100 kDa, highlighting the enzymatic breakdown of proteins during fermentation (33, 34). Our data revealed that the unfermented CM samples presented protein bands between 15 and 30 kDa, corresponding to intact, undegraded proteins, whereas the fermented samples presented a broader range of protein bands from 15 to 85 kDa, indicating partial protein hydrolysis due to microbial fermentation. Patel et al. (49) reported that unfermented camel milk presented a broader protein profile (10–315 kDa) than milk fermented with the KGL3A strain (42–124 kDa), indicating pronounced LAB-induced proteolysis.

Camel milk fermented via *KGL3A* was analyzed in 2D, revealing 31 unique protein spots ranging from 10 to 100 kDa, indicating the range of protein modifications occurring during fermentation (11). In a separate study, Khakhariya et al. (34) conducted 2D-PAGE analysis on camel and buffalo milk fermented with the *KGL4*+*WBS2A* combination. Thirty-five protein spots were identified, with 15 spots derived from buffalo milk and 20 from camel milk. These findings reveal distinct protein profiles and structural variations between the two types of fermented milk.

Structural alterations in sheep milk induced by fermentation via confocal laser scanning microscopy (CLSM). In the unfermented samples, the micrographs revealed relatively large particles, characteristic of an intact native protein matrix. In contrast, fermentation with *Lactobacillus* KGL4 triggered pronounced proteolytic activity, cleaving native proteins into smaller peptide fragments and simultaneously promoting the assembly of larger protein aggregates with more elaborate and intricate conformations. This structural reorganization was attributed to the notable polymerization potential of the Lactobacillus strain during fermentation. Furthermore, fermented sheep milk was fractionated via ultrafiltration membranes with molecular weight cutoffs of 3 and 10 kDa to obtain permeate and retentate fractions. Spectroscopic and microscopic analyses demonstrated that the 3 kDa permeate predominantly comprised low-molecular-mass peptides, whereas the 3 kDa retentate, 10 kDa (permeate and retentate), exhibited minimal variation in peptide dimensions, suggesting a relatively homogeneous size distribution within the 3–10 kDa range (35).

FTIR spectral analyses of fermented camel and Gir cow milk revealed prominent and distinct peaks in the fingerprint region (1,700–500 cm^−1^), indicative of structural changes in milk proteins during fermentation. Specifically, fermented CM presented characteristic peaks at 1,697 and 1,568 cm^−1^, corresponding to the amide I and amide II regions, respectively, suggesting proteolytic activity and peptide formation. Similarly, unique peaks in fermented Gir cow milk at 1,293 and 1,492 cm^−1^ were attributed to protein- and peptide-related vibrations. These spectral features are consistent with the observed increase in the amide I band intensity at 1,637 cm^−1^ in FCM in our study, confirming the breakdown of native proteins as well as the generation of bioactive peptides due to microbial fermentation (48).

The survival of RAW 264.7 macrophages following exposure to sheep milk fermented with the KGL4 strain was assessed via the MTT assay. The assessment involved six concentrations (0.5, 1, 2, 4, 6, and 8 mg/ml) along with a control group that did not receive the bacterial strain. At concentrations of 0.5, 1, and 2 mg/ml, fermented sheep milk demonstrated no cytotoxicity, sustaining near-complete cell viability. Conversely, higher concentrations (4, 6, and 8 mg/ml) resulted in a marked decline in viability, indicative of increased cytotoxic effects. This investigation also assessed the influence of fermented sheep milk on nitric oxide (NO) synthesis and proinflammatory cytokine expression in RAW 264.7 cells. While the LPS-stimulated group presented a pronounced increase in nitrite levels, cotreatment with fermented sheep milk significantly mitigated this increase. Notably, cells treated solely with fermented milk presented the lowest nitrite concentrations, nearly equivalent to those of the control, suggesting that fermentation-derived peptides have no detrimental effect on cellular health. Furthermore, the administration of 1 mg/ml fermented sheep milk substantially downregulated the LPS-induced increase in TNF-α, IL-6, and IL-1β, with cytokine levels closely approximating those of the control group. These findings underscore the potent anti-inflammatory capacity of fermented sheep milk (35). These findings correlate well with our study. Similar to the results reported by Pipaliya et al. (35), we also observed that cell viability significantly decreased at higher concentrations of FCM, indicating dose-dependent cytotoxicity. Additionally, our study revealed a notable reduction in the expression of proinflammatory cytokines, which aligns with previous findings. Furthermore, we also observed a substantial decrease in ROS production, further supporting the anti-inflammatory and antioxidant potential of fermented CM.

Shukla et al. (52) demonstrated that peptides derived from FCM, such as YLEELHRLNK and YLQELYPHSSLKVRPILK, formed stable interactions with human pancreatic alpha-amylase (hPAM), involving hydrogen bonds, salt bridges, and hydrophobic forces, indicating strong inhibitory potential. In our study, molecular docking revealed that the camel milk-derived peptide CCFSSCAMR interacted with human bile salt-activated lipase (hBAL), maltase-glucoamylase (hMGA), and pancreatic alpha-amylase (hPAM) through key residues such as Asp536, Gln597, Tyr208, and Ile50. These interactions, including hydrogen bonding and hydrophobic contacts, suggest that CCFSSCAMR may effectively bind within enzyme active sites, highlighting its potential as a bioactive antidiabetic peptide.

## 5 Conclusion

In conclusion, fermentation of camel milk with *Limosilactobacillus fermentum* KGL4 (MTCC 25515) with *Saccharomyces cerevisiae* WBS2A (MG101828) resulted in the development of milk with enhanced functional properties, including notable antidiabetic, antioxidant, anti-inflammatory, and proteolytic activities. After 48 h of incubation with a 2.5% inoculum, the maximum inhibition rates of α-amylase and α-glucosidase were 81.33 and 68.37%, respectively. Proteolytic activity, assessed through the OPA method, peaked at 6.41 mg/ml under these conditions. RP-HPLC profiling of the ultrafiltered fractions indicated that the 3 kDa permeate presented superior antidiabetic potential, whereas the 10 kDa retentate presented the highest antioxidant capacity. Protein degradation was confirmed by SDS–PAGE and 2D gel electrophoresis, which revealed 33 discrete spots between 15 and 70 kDa, suggesting substantial proteolysis. FTIR analysis demonstrated an increase in β-sheet structures, and CLSM imaging revealed a decrease in aggregate size, reflecting changes in protein organization due to fermentation. One of the peptides identified, CCFSSCAMR, showed strong binding affinity to the human digestive enzymes hBAL, hPAM, and hMGA, as determined by molecular docking. Furthermore, anti-inflammatory analysis of LPS-induced RAW 264.7 macrophages revealed decreased levels of TNF-α, IL-6, and IL-1β; reduced ROS generation; and minimal apoptotic cell death. These findings highlight the therapeutic potential of fermented camel milk as a rich source of multifunctional bioactive peptides, supporting its further investigation in clinical and nutraceutical applications. While this study demonstrated the multifunctional potential of fermenting camel milk through LAB with yeast cofermentation, several aspects remain to be explored to increase its translational relevance. Future research should focus on *in vivo* validation of antidiabetic, antioxidant, and anti-inflammatory effects, along with clinical trials, to confirm the efficacy of these treatments and establish optimal dietary dosages. The bioavailability, gastrointestinal stability, and metabolic fate of the identified bioactive peptides need investigation to better understand their functional performance under physiological conditions. Additionally, large-scale fermentation, shelf-life studies, and evaluation of strain–strain interactions under various fermentation conditions are necessary to ensure product stability, scalability, and consistent peptide bioactivity. Addressing these aspects will provide a stronger foundation for developing fermented camel milk as a scientifically validated functional food or nutraceutical ingredient.

## Data Availability

The original contributions presented in the study are included in the article/supplementary material, further inquiries can be directed to the corresponding author.
