# Supplementary material for: Peptidomic profiling and molecular dynamics study of bioactive peptides from fermented camel milk: considering the fermentation time dependent proteolysis by Lactobacillus and Saccharomyces with antidiabetic, antioxidative and anti-inflammatory activities
Source: Front Nutr. 2026 Mar 10;13:1709521. doi: 10.3389/fnut.2026.1709521 (PMC13008740; doi:10.3389/fnut.2026.1709521)
Supplement: Supplementary file 1 [file Data_Sheet_1.docx]

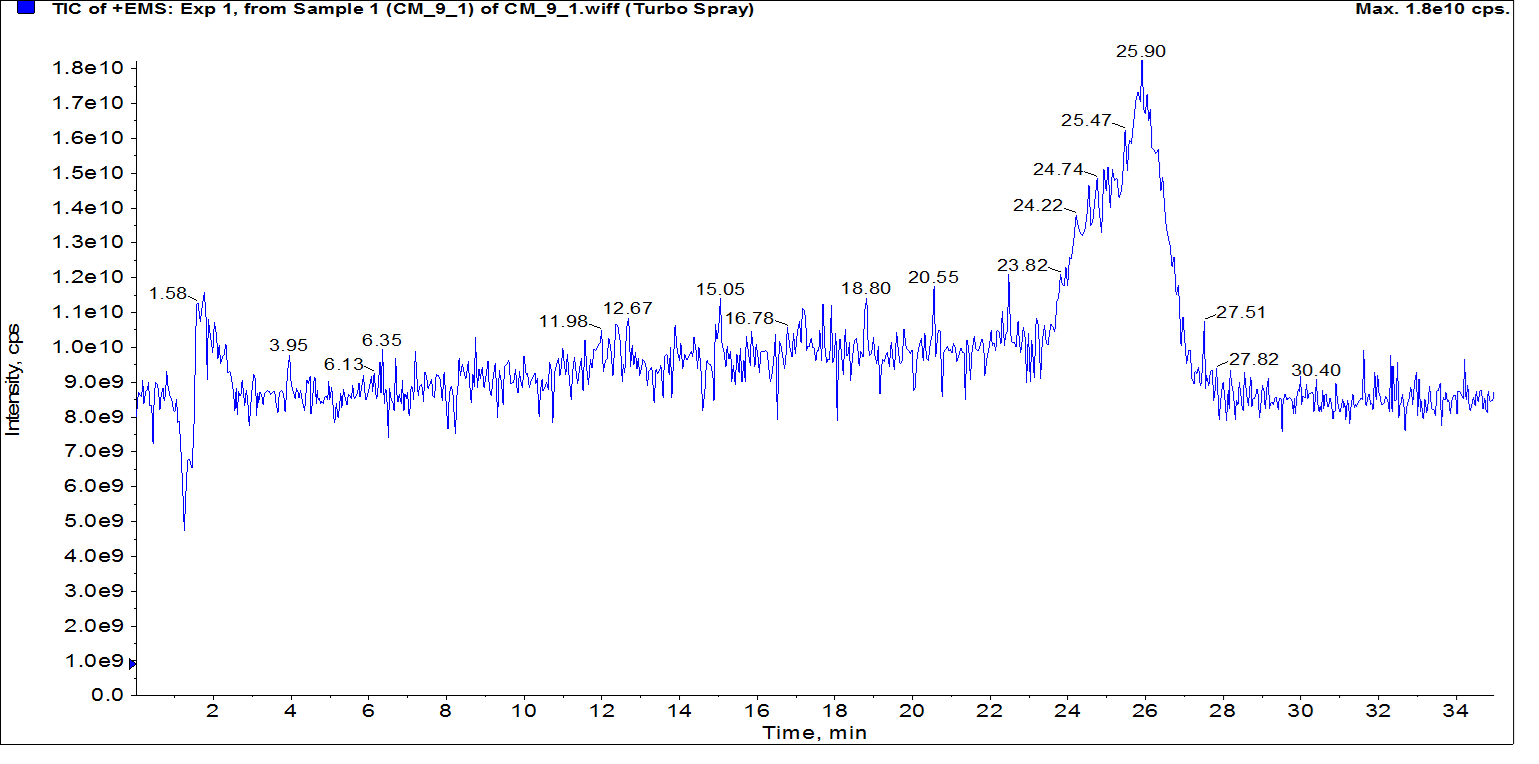
 Figure S1: The total ion chromatogram of fermented camel milk with M9+WBS2A (EMS to EPI scan in LC-MS)


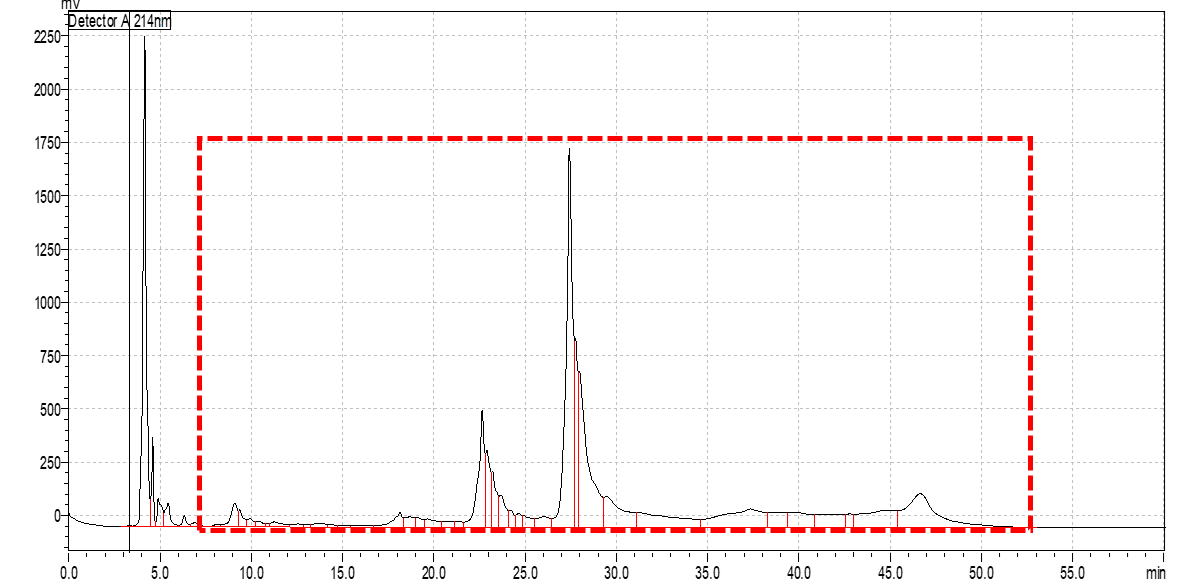


Figure S2: RP-HPLC chromatogram of unfermented camel milk


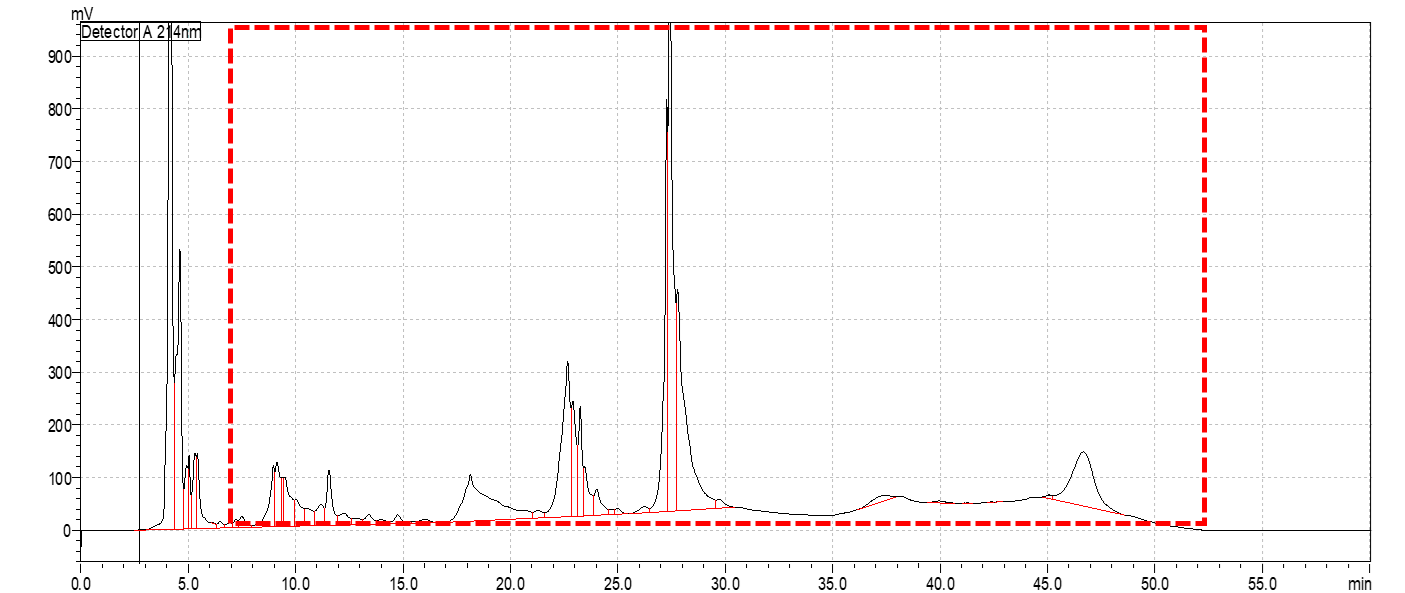
Figure S3: RP-HPLC chromatogram of camel milk fermented with M9+WBS2A


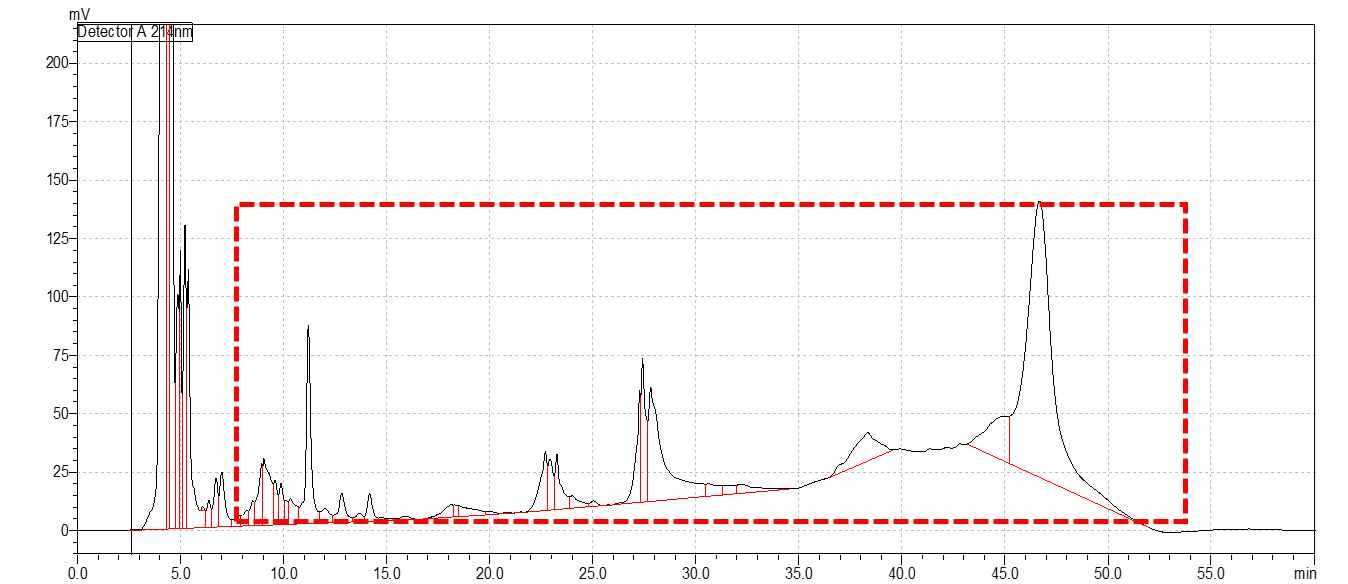


Figure S4: RP-HPLC chromatogram of 3 kDa permeate from camel milk fermented with M9+WBS2A


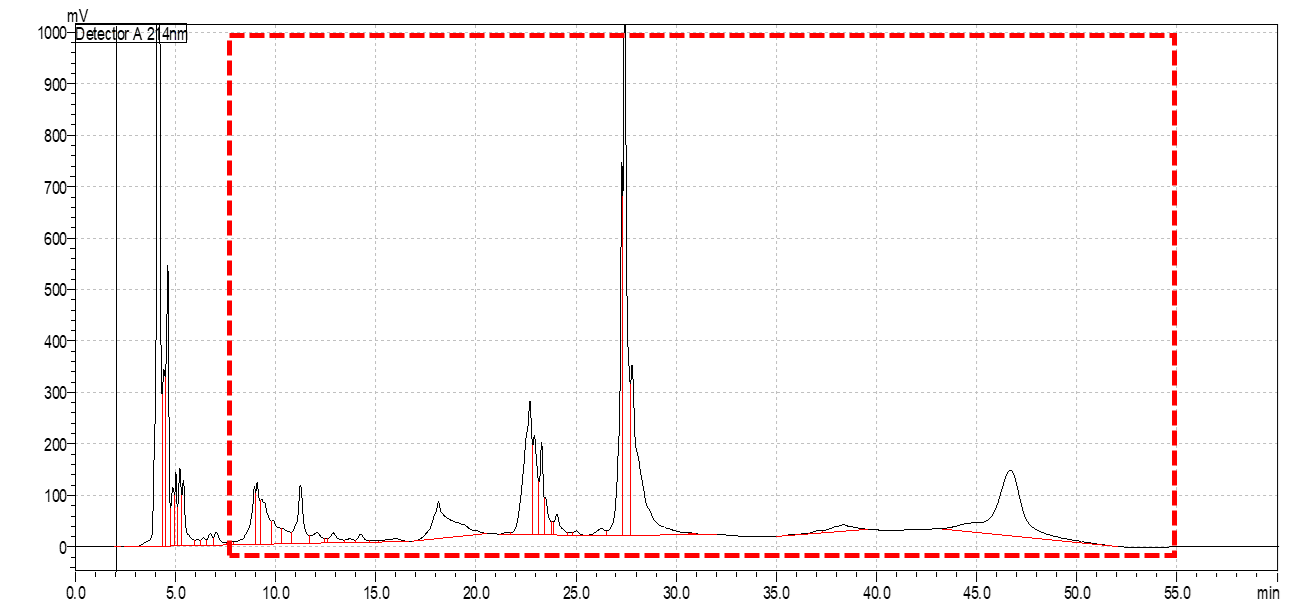
Figure S5: RP-HPLC chromatogram of 3 kDa retentate from camel milk fermented with M9+WBS2A


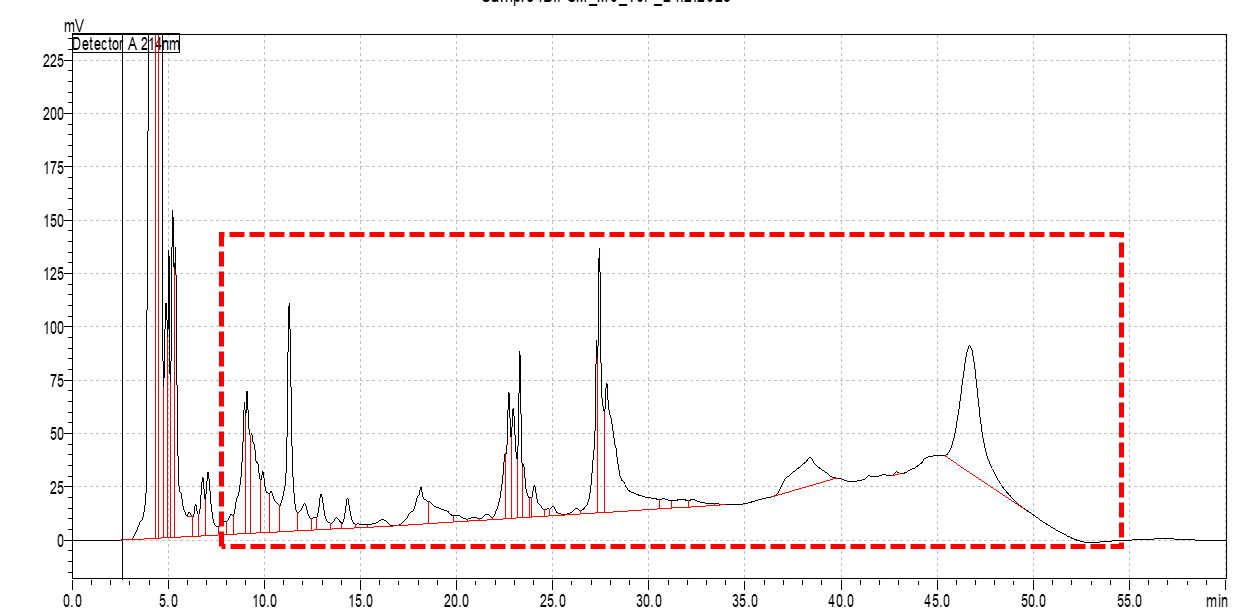


Figure S6: RP-HPLC chromatogram of 10 kDa permeate from camel milk fermented with M9+WBS2A


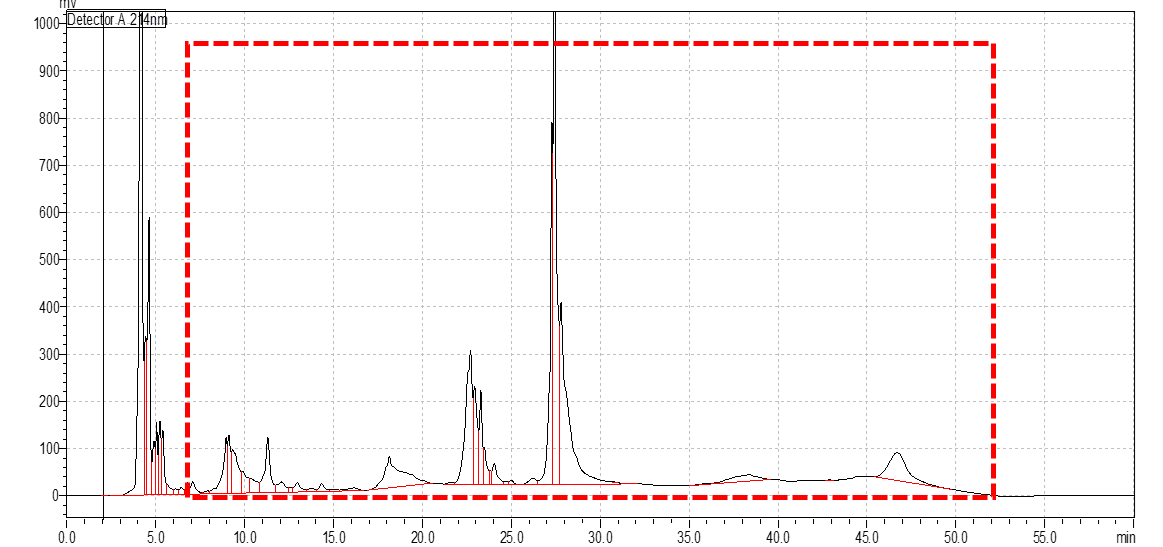
Figure S7: RP-HPLC chromatogram of 10 kDa retentate from camel milk fermented with M9+WBS2A

|  | | | |
| --- | --- | --- | --- |
| 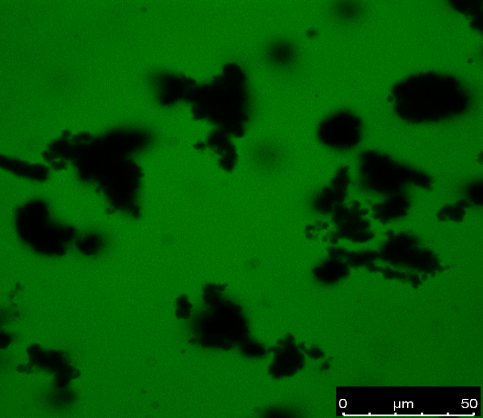 | 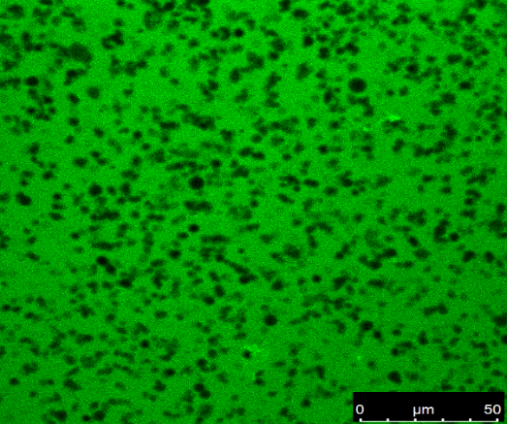 | | 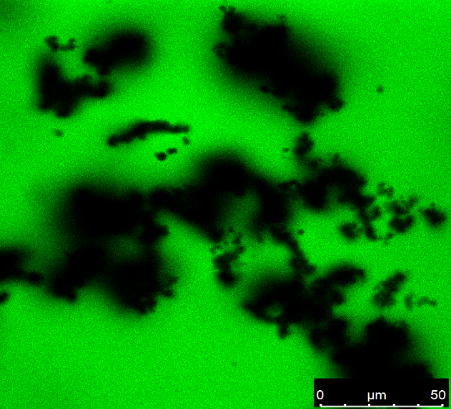 |
| **Unfermented camel milk** | **Fermented camel milk** | | **3 KDa Permeate** |
| 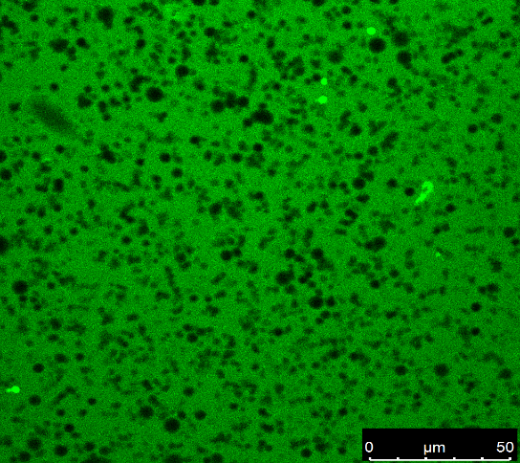 | 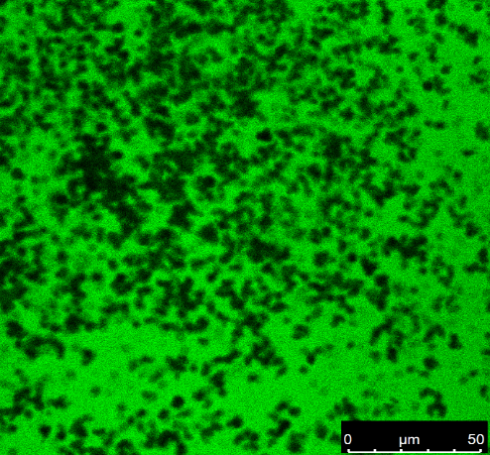 | 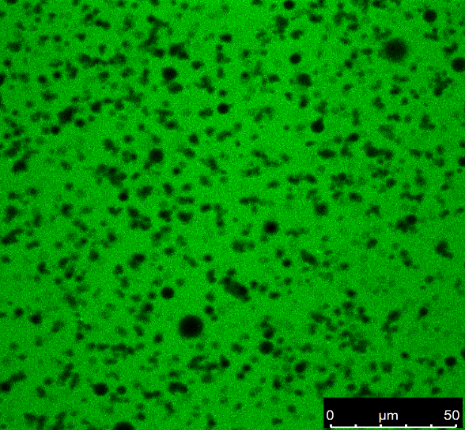 | |
| **3 KDa Retentate** | **10 KDa Permeate** | **10 KDa Retentate** | |

Figure S8: CLSM images showing the microstructure of fermented camel milk using M9+WBS2A
